# 1,2 Intercompartmental Supraretinacular Artery-Based Vascularized Graft for Scaphoid Nonunion With Avascular Necrosis

**DOI:** 10.7759/cureus.11290

**Published:** 2020-11-01

**Authors:** Terrence Jose Jerome, Ramesh Prabu, Thirumagal Kuppusamy Terrence, Suresh Bhalaji, Bhuvaneswari Shanmugasundaram

**Affiliations:** 1 Orthopaedics, Hand and Reconstructive Microsurgery, Olympia Hospital and Research Centre, Trichy, IND; 2 Orthopaedics and Traumatology, KAP Vishwanatham (KAPV) Medical College Hospital, Mahatma Gandhi Memorial (MGM) Hospital, Trichy, IND; 3 Reproductive Medicine/Obstetrics and Gynecology, Olympia Hospital and Research Centre, Trichy, IND; 4 Urology, Dhanalakshmi Medical College, Perambalur, IND; 5 Pharmacology, Dhanalakshmi Medical College, Perambalur, IND

**Keywords:** 100% union, good outcome, scaphoid nonunion, avascular necrosis, 1, 2 icsra based vascularized graft, dorsal on lay

## Abstract

Background

The treatment for scaphoid nonunion with avascular necrosis is vascularized and non-vascularized bone grafts. A vascularized bone graft promotes biological healing and revascularizes ischemic bone. The purpose of this retrospective study is to analyze the outcome of 1,2 intercompartmental supraretinacular artery (1,2-ICSRA)-based vascularized graft in scaphoid nonunion with avascular necrosis.

Materials and methods

We treated 11 patients with scaphoid nonunion with avascular necrosis using a (1,2-ICSRA)-based vascular graft and Herbert screw fixation between 2013 and 2017. Plain radiographs, computed tomography (CT) scan, magnetic resonance imaging (MRI) confirmed the avascular necrosis in all patients. We noted the age, delay in treatment, time for bone union, preoperative range of movements, grip strength, scapholunate, intrascaphoid angle, and radiolunate angles. We confirmed the bone union by CT scan and measured the functional outcome with pain score, modified Mayo wrist score, grip strength, range of movement, and Disabilities of the Arm, Shoulder, and Hand (DASH) score.

Results

The mean age of the patients was 29 years (range 20-42 years). The mean follow-up was 31 months (range, 26-36 months). All patients achieved good radiological union and revascularization of the proximal pole necrosis at an average of 14 weeks (range, 12-18 weeks). There was a significant postoperative improvement in grip strength, visual analog scale VAS score, intrascaphoid angle, scapholunate angle, and radiolunate angle (p<.05). The mean range of wrist flexion was 88%, extension 70%, radial deviation 80%, and ulnar deviation 85% of the opposite side.

Conclusions

Scaphoid nonunion with avascular necrosis can be treated with a 1,2-ICSRA-based vascularized bone graft. Vascularized bone grafts promote biological healing and revascularization of the ischemic bone.

## Introduction

Acute scaphoid fractures fail to unite in 5% to 15% of cases [1]. These scaphoid nonunions usually remain asymptomatic initially and have demonstrated a uniform progression to degenerative carpal arthritis and scaphoid nonunion advance collapse (SNAC) overtime with pain, restriction of movements, and activities [2-4]. The duration of scaphoid nonunion, delay in diagnosis, inadequate immobilization, proximal pole involvement, fracture instability and displacement, avascular necrosis, and associated carpal instability are the risk factors associated with scaphoid nonunion [5].

The disruption of blood supply from the distal to the proximal pole scaphoid following a fracture predisposes the scaphoid to ischemia and avascular necrosis [6]. The treatment should be efficacious and meticulously planned because the proximal pole vascularity correlates with the success and the scaphoid union.

The purpose of the study was to review a single surgeon’s experience with scaphoid nonunion and avascular necrosis treated by a 1,2 intercompartmental supraretinacular artery (1,2-ICSRA)-based vascularized bone graft.

## Materials and methods

Patients, setting, and ethics

The institutional ethical committee review board approved this retrospective study. We operated on 11 consecutive patients with scaphoid nonunion and avascular necrosis using a 1,2-ICSRA based vascularized bone graft (VBG) between 2013 and 2017. We obtained informed consent from the patients for the pictures. The side involved, gender, smoking history, age at the time of injury, presence of pain, range of motion (wrist flexion, extension, radial deviation, and ulnar deviation), and grip strength were noted. 

**Figure 1:**
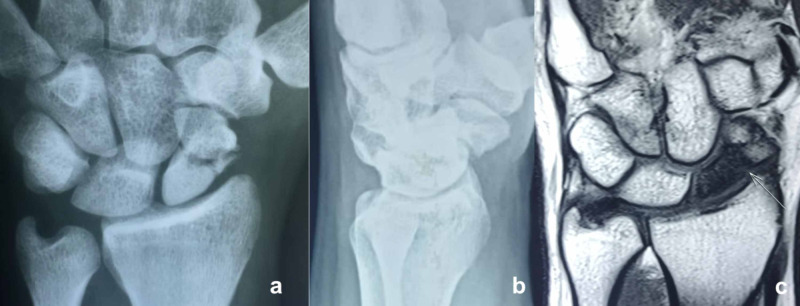
Posteroanterior (a) and lateral radiographs (b) of a 29-year-old man with left side scaphoid nonunion and avascular necrosis of 36 months duration. MRI (c) showing scaphoid nonunion with a T1-weighted coronal image showing diffusely decreased signal within the proximal pole We identified no bleeding at the surgery.

Plain radiographs (posteroanterior, lateral, and oblique wrist views, and a posteroanterior ulnar deviation view) with sclerosis, fragmentation, and collapse confirmed the diagnosis. Computed tomography (CT) scans effectively confirmed the collapse with nonunion bone characteristics such as resorptive changes, sclerosis, joint space narrowing, and fragmentation. Magnetic resonance imaging (MRI) with low-signal intensity on T1-weighted sequences, lack of contrast enhancement, and homogenously decreased signal on T2-weighted fat-suppressed images confirmed the avascular necrosis of the proximal pole of the scaphoid (Figure 1).

In addition, we noted the preoperative scapholunate, lateral intrascaphoid angle, and radiolunate angles in all patients. We excluded associated carpal fractures, dislocations, and the presence of concomitant distal radius fractures from the study. The time interval between the injury and surgery was noted.

Technique

**Figure 2:**
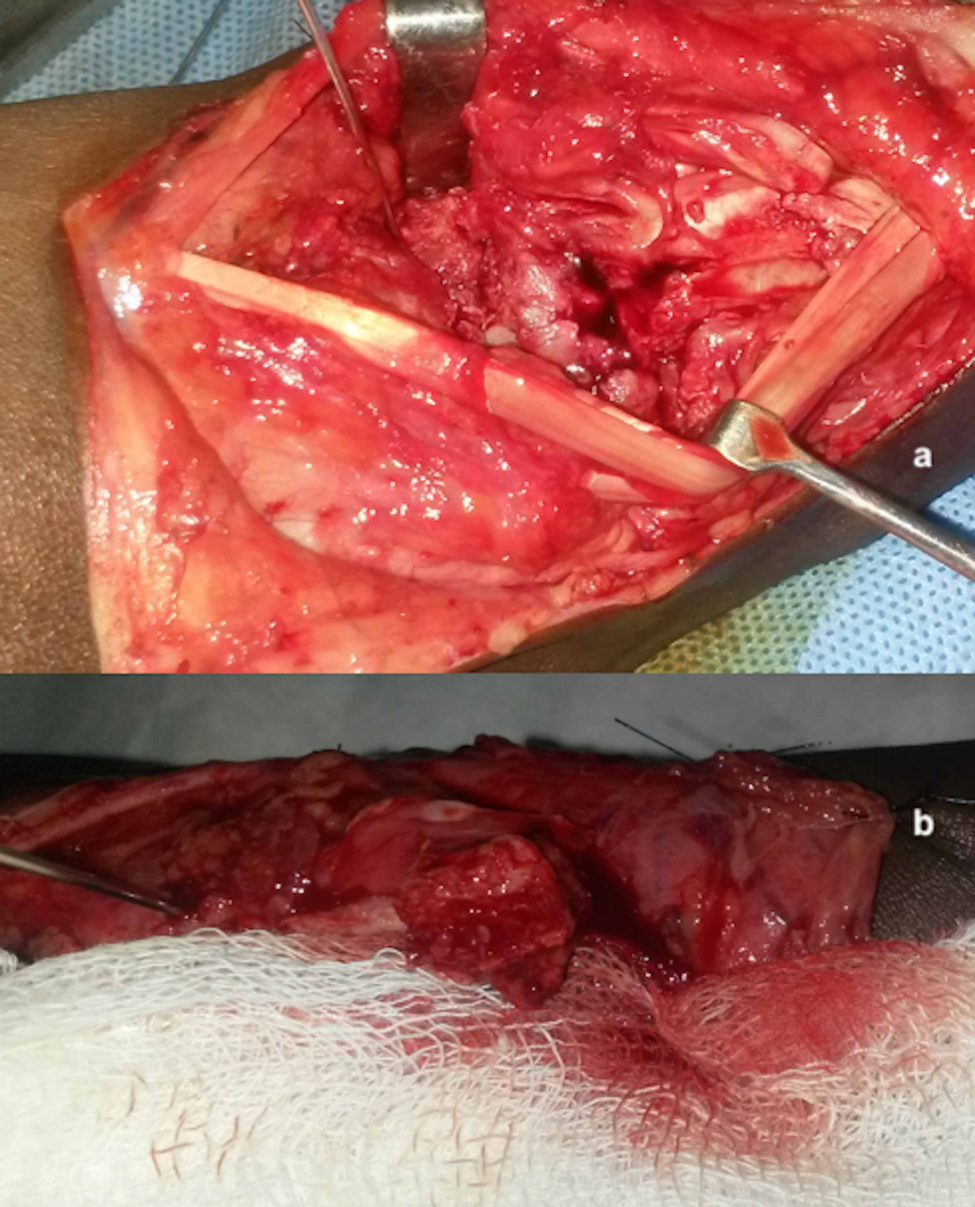
Intraoperative pictures (a) Absence of punctate bleed from the proximal scaphoid pole, (b) 1,2-ICSRA-based VBG with active bleeding VBG: vascularized bone graft

All patients were operated on under a supraclavicular brachial block with a tourniquet in the arm inflated. We performed the surgical technique as described in previous studies [7]. We made a curvilinear dorsoradial incision over the wrist. The 1,2-ICSRA pedicle was identified between the first and second extensor compartments and elevated from the radius. We exposed the scaphoid nonunion with avascular necrosis site and debrided and curetted the fibrous tissue and sclerotic bone within the nonunion site, preserving the cortical bone as much possible. The absence of punctate bleeding in the proximal pole confirmed the diagnosis of avascular necrosis (Figure 2).

We prepared a trough over the nonunion site for the dorsal inlay of the 1,2-ICSRA graft. We harvested a sufficiently large 1,2-ICSRA graft with the vessel, cuff of the retinaculum, from the radius with an osteotome and chisel, 1.5 cm proximal to the radiocarpal joint. The tourniquet was released to assess the bleeding from the VBG. We then transposed the VBG to the nonunion site trough as dorsal on-lay. We reduced the scaphoid nonunion site using a joystick technique and compressed it with a headless compression screw. The 1,2-ICSRA graft dorsal inlay graft was stabilized to the scaphoid nonunion trough with a closed-cut Kirschner wire. A scaphocapitate wire was added in very few cases to augment fixation and immobilization.

Postoperative care

All patients were given a thumb cast for six weeks and changed to a short arm splint, which the patient used it as a supportive splint between the therapy. Radiographs were done at the monthly interval to assess the fracture healing status. CT scan was done between eight and 16 weeks to confirm the union and evaluate the avascular necrosis progress of the scaphoid.

Follow-up

Bridging the trabecula crossing, the fracture site was considered as the union in radiographs and the CT scan. We measured the lateral Intrascaphoid angle, scapholunate angle (degree), and radiolunate angles. (intrascaphoid angle <45 degree, radiolunate angle (-10 to 12 degree), scapholunate angle (30 to 60 degrees), normal values). We considered a radiolunate angle >15 degrees as a dorsal intercalated segment instability (DISI) deformity. Also, the active wrist range of motion and grip strength was measured using a goniometer and Jamar® hydraulic hand dynamometer (Model J00105; Sammons Preston, Bolingbrook, Illinois). The pain was assessed on a visual analog scale (VAS) and function by the Quick Quick DASH questionnaire (0: no limitation, 100: maximum limitation) and the Mayo wrist score (MWS; 91-100: excellent; 80-90: good; 65-79: fair; and < 65: poor).

Statistics 

 A paired t-test determined statistical significance between preoperative and postoperative measurements. Two-tailed p<0.05 was considered significant.

## Results

Patient and nonunion characteristics

The mean age of the patients was 29 years (range 20-42 years.) There were nine male and two female patients (Table 1).

**Table 1:** Demographics and follow-up

Patient No	Age	Side	Gender	Smoker	Mode of injury	Initial treatment	Time interval injury-surgery (months)	Time interval surgery – bony union (weeks)	Wrist Score: (Mod. Mayo)	VAS score	DASH score	Follow-up (months)	Complications
1	38	Right	M	No	Fall	conservative	9	17	80	1	3.3	26	nil
2	28	Left	M	No	Motorcycle accident	neglected	12	14	80	0	8.3	28	nil
3	26	Left	M	Yes	Fall	neglected	14	13	85	0	9.2	36	nil
4	30	Right	M	Yes	Fall	conservative	15	12	85	0	3.3	32	nil
5	20	Right	F	No	Motorcycle accident	conservative	10	15	85	0	6.7	36	nil
6	22	Right	F	Yes	Fall	conservative	14	12	85	0	3.3	28	nil
7	27	Right	M	No	Fall	conservative	12	13	80	1	8.3	27	pin irritation
8	20	Right	M	Yes	Fall	neglected	18	12	80	0	5.8	32	nil
9	29	Left	M	No	Fall	conservative	36	14	80	0	3.3	34	nil
10	35	Right	M	No	Motorcycle accident	conservative	48	13	85	0	5.8	33	nil
11	42	Right	M	Yes	Fall	conservative	40	18	80	0	3.3	32	nil

Eight of the 11 patients had right wrist involvement. The most common mechanism of injury was a fall on the outstretched hand (n=8). Five patients were smokers of > 10 years duration. There were no open injuries or associated neurovascular lesions. Eight patients had an initial thumb cast, and three had no treatment for the scaphoid fractures. The median time from the injury to surgery was 20.7 months (range, 9-48 months). 

Outcomes

**Figure 3:**
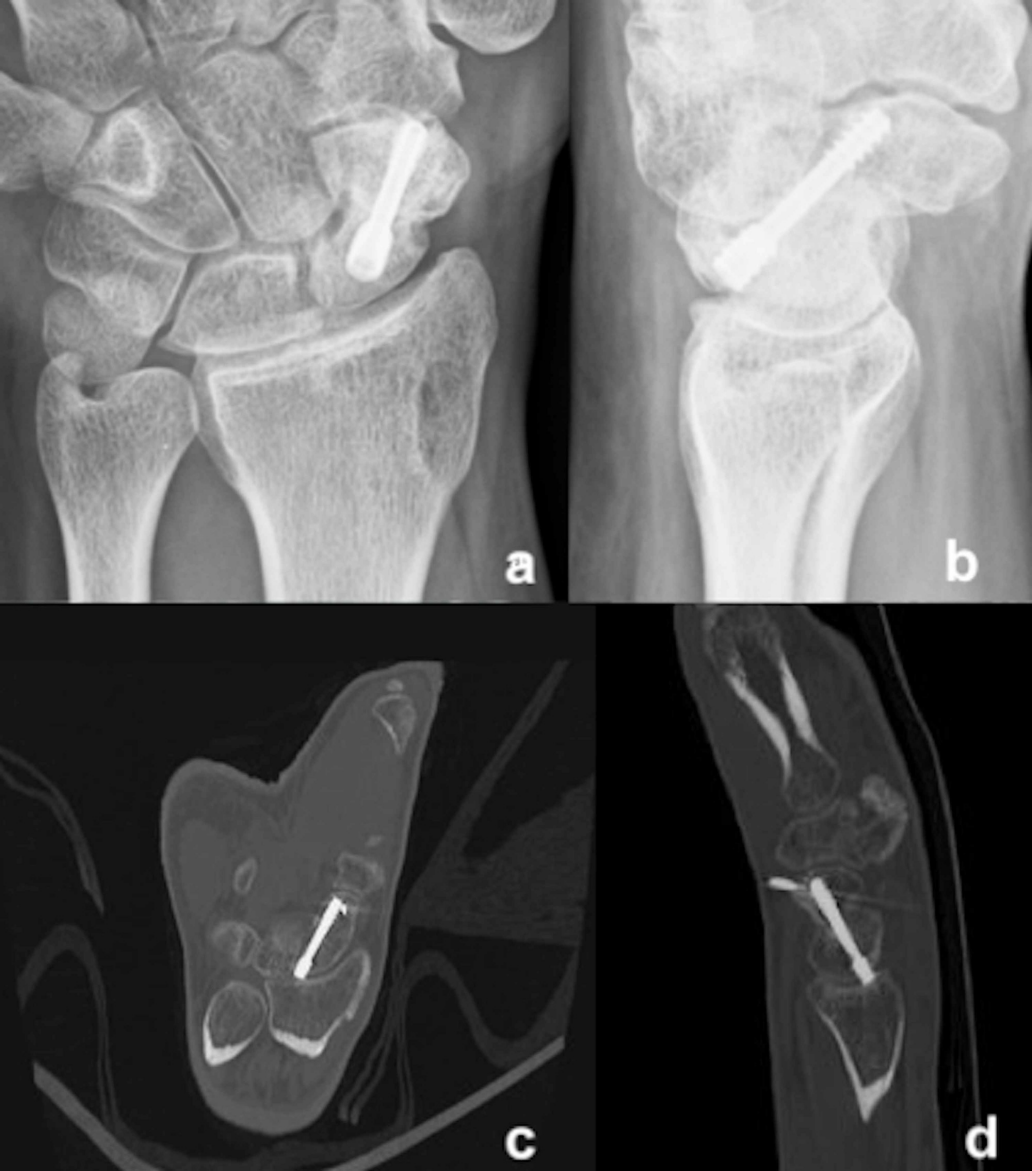
Posteroanterior (a) and lateral (b) radiographs showing good union at weeks after the surgery, CT scan (c, d) shows good union with vascularized bone graft incorporation at the nonunion site

The mean follow-up was 31 months (range, 26-36 months). All patients achieved good radiological union and revascularization of the proximal pole necrosis at an average of 14 weeks (range, 12-18 weeks) (Figure 3).

One patient had Kirschner wire irritation, which settled later with pin removal (Table 2).

**Table 2:** Postoperative functional outcomes SD - standard deviation; VAS - visual analog score; RL - radiolunate; SL - scapholunate; IS - intrascaphoid

Outcomes	Preoperative (Mean (SD))	Postoperative (Mean (SD))	P-value
Grip strength	74(3.7)	96.2(2.0)	P<0.05
VAS	5.0(1.0)	0.1(0.4)	p<0.05
IS angle	70.6(3.4)	35.0(2.9)	p<0.05
SL angle	86.2(2.6)	47.4(2.4)	p<0.05
RL angle	13.9(5.2)	2.8(3.2)	p<0.05

None of the patients had nonunion or graft failures. There was a significant postoperative improvement in grip strength, VAS score, intrascaphoid angle, scapholunate angle, and radiolunate angle (p<.05). The mean range of wrist flexion was 88%, extension 70%, radial deviation 80%, and ulnar deviation 85%, of the opposite side. The median VAS pain score was 0.1 (range, 0-1). Median DASH score was 5.5 (range, 3.3-9.2), and Mayo wrist score 82.5 (range, 80-85).

## Discussion

There is little consensus about treatment options for proximal pole avascular necrosis (AVN), which occurs in 13% to 50% of scaphoid fractures [8]. Also, there is controversy about the use of vascular grafts and non-vascular grafts in scaphoid nonunion [6,9]. Various authors have reported good to excellent results for scaphoid nonunion results using vascularized grafts such as 1,2-ICSRA grafts, volar carpal artery grafts, free tricortical iliac crest grafts, and medial femoral condyle grafts [10]. They recommend VBGs for proximal pole AVN, arguing poor healing potential with nonvascularized bone grafts. VBG follows the biological principle to promote healing by significantly increasing bone blood flow, raising high levels of osteoid-covered and osteoblast covered trabecular surfaces [10]. Also, VBG uses de novo vascularity to enhance the creeping substitution of in situ necrotic bone [10].

The clinical presentation and diagnosis of scaphoid nonunion with AVN are variable. In our study, clinical findings, pain restriction of wrist dorsiflexion, and MRI findings of low-signal intensity on T1-weighted sequences, lack of contrast enhancement, and homogenously decreased signal on T2-weighted fat-suppressed images were consistent in our scaphoid nonunions with avascular necrosis. The absence of intraoperative punctate bleeding is considered as a reference standard for AVN confirmation despite shortcomings. All our patients had an absence of intraoperative punctate bleeding. Furthermore, we recommend that histological evaluation should be kept as one of the criteria for the assessment of AVN because it can represent the true presence of osteoblasts, remodeling potential, necrotic myeloid tissue, and changes in the trabecular surface.

Zaidemberg et al. reported a 100% union in 11 patients treated with 1,2-ICSRA pedicled VBG and Kirschner wire. None of the patients had AVN [11]. Boyer et al. noted 60% union in their series of scaphoid nonunion with AVN in 10 patients treated with a Herbert screw and Kirschner wires at an average of 4.6 months [12]. Straw et al. reported 22% union in their series of 22 patients between eight and 16 weeks duration using a mini-Herbert screw and Kirschner wires [13].

Chang et al. found a 71% union in 24 patients with AVN at an average of 15.6 weeks [14]. They reported graft extrusion, superficial and deep infections, and instrumentation failures as complications in their study. Lim et al. reported a 86% union using 1,2-ICSRA pedicled VBG and Kirschner wire fixation in the scaphoid with AVN at an average of 14 weeks [15]. Morris et al. noted a 100% union in their 11 patients with AVN treated with a headless compression screw at an average of 11.4 weeks [16].

Our study noted 100% union in our series of 11 patients treated with 1,2-ICSRA pedicled VBG as dorsal only graft and Herbert screw fixation. We stabilized the graft with Kirschner wire. CT scan confirmed the union at an average of 14 weeks. In addition to the union, all patients achieved a normal scaphoid geometry with the vascularized structural graft provided by the 1,2-ICSRA and had normal carpal kinematics. There was significant (p<.05) improvement in the range of movements, grip strength, and pain in the follow-up. None of the patients had graft displacement, nonunion, or progression of AVN to degenerative arthritis and scaphoid nonunion advance collapse.

An overall literature review found 76% AVN based on absent bleeding, MRI, radiographs, and/or CT scans [10]. Our noted radiographs, CT scan, MR findings, and the absence of intraoperative punctate bleeding in all of our patients confirming 100% sensitive and specific AVN. The literature review found union in 77% of the patients operated with 1,2-ICSRA-based VBG [10]. Irrespective of the proximal pole size, the dorsal on-lay 1,2-ICSRA-based VBG graft produced a 100% union in our small cohort.

All patients achieved radiological union, with a significant improvement in radiolunate, scapholunate, and lateral intrascaphoid angle (p<.05). None of the patients had a scaphoid humpback, fragmentation, scapholunate widening, or arthritis. Chang et al. noted delayed union in smokers (average 21 weeks) as compared with non-smokers (average 15 weeks) [14]. In our study, we did not find any delay in union between smokers and non-smokers. Smoking was not a risk factor for the union. We saw pin irritation in one patient, which settled with removal.

Our studies' limitations are the small sample size, retrospective design, and lack of a control group. VBG is technically demanding, expensive, requires more operative time than non-vascularized bone grafts, and carries the risk of donor site morbidity. Moreover, there are no absolute indications for VBG in these cases.

Various studies and metanalysis have reported an 88% union rate with vascularized bone grafting of scaphoid nonunions with AVN as compared to 47% with conventional bone grafting. Our study documented that using 1,2-ICSRA-based VBG in scaphoid nonunion with AVN produced 100% union by improving the rate of and time required for healing and revascularizing the ischemic scaphoid proximal pole. We need a large prospective study and a controlled study to analyze the standard treatment modalities in such cases.

## Conclusions

Given the scenario, with no consensus in diagnosis and on which treatment modality predicts favorable outcome, the 1,2-ICSRA pedicled vascularized graft in a single surgeon’s experience produced excellent bone union and achieved biological healing. AVN with necrosis, structural defects, and proximal pole fragment sizes benefit from 1,2-ICSRA vascularized bone grafting.
